# BET Bromodomains Regulate Transforming Growth Factor-β-induced Proliferation and Cytokine Release in Asthmatic Airway Smooth Muscle*

**DOI:** 10.1074/jbc.M114.612671

**Published:** 2015-02-19

**Authors:** Mark M. Perry, Andrew L. Durham, Philip J. Austin, Ian M. Adcock, Kian Fan Chung

**Affiliations:** From the Experimental Studies, National Heart and Lung Institute, Imperial College London and Royal Brompton National Institute for Health Research Biomedical Research Unit, London SW3 6LY, United Kingdom

**Keywords:** Asthma, Bromodomain-containing Protein 4 (BRD4), Cell Proliferation, Interleukin 6 (IL-6), Myc (c-Myc), Airway Smooth Muscle, CXCL8, I-BET762, JQ1/SGCBD01

## Abstract

Airway smooth muscle (ASM) mass is increased in asthma, and ASM cells from patients with asthma are hyperproliferative and release more IL-6 and CXCL8. The BET (bromo- and extra-terminal) family of proteins (Brd2, Brd3, and Brd4) govern the assembly of histone acetylation-dependent chromatin complexes. We have examined whether they modulate proliferation and cytokine expression in asthmatic ASM cells by studying the effect of BET bromodomain mimics JQ1/SGCBD01 and I-BET762. ASM cells from healthy individuals and nonsevere and severe asthmatics were pretreated with JQ1/SGCBD01 and I-BET762 prior to stimulation with FCS and TGF-β. Proliferation was measured by BrdU incorporation. IL-6 and CXCL8 release was measured by ELISA, and mRNA expression was measured by quantitative RT-PCR. ChIP using a specific anti-Brd4 antibody and PCR primers directed against the transcriptional start site of *IL-6* and *CXCL8* gene promoters was performed. Neither JQ1/SGCBD01 nor I-BET762 had any effect on ASM cell viability. JQ1/SGCBD01 and I-BET762 inhibited FCS+TGF-β-induced ASM cell proliferation and IL-6 and CXCL8 release in healthy individuals (≥ 30 nm) and in nonsevere and severe asthma patients (≥100 nm), with the latter requiring higher concentrations of these mimics. JQ1/SGCBD01 reduced Brd4 binding to *IL8* and *IL6* promoters induced by FCS+TGF-β. Mimics of BET bromodomains inhibit aberrant ASM cell proliferation and inflammation with lesser efficiency in those from asthmatic patients. They may be effective in reducing airway remodeling in asthma.

## Teaser

**Background:** Airway smooth muscle (ASM) from asthmatics is in a hyperproliferative state and releases more cytokines than healthy individuals.

**Results:** Inhibition of BET bromodomains reduces ASM proliferation and cytokine release by reducing Brd4 binding to the promoter regions of IL-6 and CXCL8.

**Conclusion:** BET bromodomain mimics inhibit aberrant ASM proliferation and inflammation in asthmatic patients.

**Significance:** These compounds may reduce airway remodeling in asthma.

## Introduction

Chronic airflow obstruction and airway inflammation and remodeling are fundamental characteristics of asthma (1). Airway smooth muscle (ASM)^4^ mass is increased in asthma because of enhanced ASM hyperplasia and hypertrophy, resulting in subepithelial fibrosis and narrowing of the airways. ASM cells also play a key role in the chronic inflammatory response in the airways of asthmatics through the expression of cytokines, growth factors, and proteases (2), which can induce phenotypic changes in ASM (3). TGF-β promotes ASM cell proliferation and size (4) and also induces the release of IL-6 and CXCL8, which are involved in many inflammatory effects including ASM-induced mast cell activation and the release of CCL11 and vascular endothelial growth factor (5–7).

Severe asthma is characterized by the persistence of symptoms despite optimal therapy with corticosteroids (8, 9). ASM mass and subepithelial fibrosis in the bronchial airways is increased in severe asthma patients when compared with nonsevere asthma patients (10, 11). This may be a consequence of the enhanced proliferation of ASM cells from patients with severe asthma compared with those from nonsevere asthma following stimulation with FCS and TGF-β (12–14). In addition to an abnormal proliferative response, these cells also demonstrate relative corticosteroid insensitivity (15), and because these responses are seen after up to five cell culture passages, it is likely that they are due to altered or rewired intracellular signaling pathways or to epigenetic mechanisms (16). Reduced responses of blood monocytes and alveolar macrophages to corticosteroids representing corticosteroid insensitivity have also been reported in patients with severe asthma (17, 18).

Histone modifications such as acetylation, phosphorylation, and methylation are regulated in a temporal and spatial manner to control chromatin structure under a “histone code or language” (19, 20). Enhanced histone acetylation is associated with altered cellular function including the increased expression of inflammatory genes. Acetylated histones are detected by proteins that contain a bromodomain motif, such as the BET (bromo- and extra-terminal) family members that include Brd2, Brd3, and Brd4 and that govern the assembly of histone acetylation-dependent chromatin complexes. This epigenetic tag is linked to control RNA polymerase II activity via recruitment of transcriptional activators such as pTEFb (21, 22). BET family members such as Brd4 have been implicated in the regulation of NF-κB-induced inflammatory gene expression in monocytes and macrophages through recognition of acetylated histone tags (23, 24). BET proteins can also associate with c-Myc to regulate cell proliferation (24).

We hypothesized that BET proteins play an important role in the regulation of proliferation and release of pro-inflammatory cytokines in ASM cells from patients with asthma. BET mimics JQ1/SGCBD01 and I-BET762 that prevent the association of bromodomain-containing proteins with acetylated lysines have recently been developed (23, 25). These mimics affect proliferation and the expression of some inflammatory genes in murine and human cell types (23, 25, 26), but this is the first time that effects have been reported in these primary ASM cells from patients with asthma. We used these inhibitors to investigate the role of BET proteins in the regulation of TGF-β-induced proliferation and inflammation of ASM obtained from patients with different severities of asthma. Our results show that inhibition of BET bromodomains can reduce both aberrant ASM cell proliferation and expression of cytokines from patients with severe asthma by reducing Brd4 binding to the promoter regions of *IL8* and *IL6* genes.

## MATERIALS AND METHODS

### 

#### 

##### Patient Recruitment

Subjects were defined as nonasthmatic on the basis of absent history of asthma and a normal responsiveness of the airways to the constrictor effect of metacholine measured as PC20 > 16 mg/ml and nonsevere or severe asthmatic according to American Thoracic Society workshop definition of severe refractory asthma (27). Current smokers and former smokers with a greater than 5 pack-year history were excluded. Subject characteristics are shown in Table 1. Bronchial biopsies were obtained from segmental and subsegmental airways of the right lower lobe by fiberoptic bronchoscopy as described previously (11). The study conformed to the Declaration of Helsinki and was approved by the ethics committee of the Royal Brompton Hospital. Written informed consent was obtained from each subject.

**TABLE 1:** **Clinical characteristics of subjects** The data are shown as means ± S.E. BDP, beclomethasone dipropionate; FEV_1_, forced expiratory volume in 1 s; FVC, forced vital capacity; PC_20_, provocative concentration of methacholine causing a 20% fall in FEV_1_; NA, not available; CS, corticosteroid.

	No asthma	Nonsevere asthma	Severe asthma
*n*	9	9	9
Age (years)	36.4 ± 12.7	42.4 ± 16.2	40.9 ± 11
Sex (male/female)	7/2	6/4	3/6
Duration of asthma (years)	NA	22.2 ± 16.8	25.6 ± 13.2
Atopy (*n*)*^a^*	0	8	7
Inhaled CS (μg of BDP equivalent)	0	0	1688.9 ± 176.4
On daily oral CS (*n*)	0	0	3
FEV_1_ (liters)	4.0 ± 0.5	2.8 ± 0.7	2.7 ± 0.8
FEV_1_ (% predicted)	104.2 ± 7.3	84.7 ± 12.3	84.5 ± 18.3
β-Agonist reversibility (%)*^b^*	NA	12.3 ± 11.9	19.5 ± 14.6
PC_20_ (mg/ml)	>16	0.69 ± 0.64	0.20 ± 0.39

*^a^* Defined as positive skin prick tests to one or more common aeroallergens.

*^b^* Measured as percent increase in FEV_1_ after 400 μg of salbutamol.

##### ASM Cell Culture and Stimulation

ASM cells were isolated and cultured from bronchial biopsies as previously described (4, 15). At confluence, ASM cell cultures exhibited a typical “hill and valley” appearance. Human airway smooth muscle cells at passages 3–4 from nine different donors were used. Prior to treatment, ASM cells were plated onto 96-well plates for measurement of DNA synthesis and cytokine release, and 6-well plates for RNA and protein extraction. Cells were growth-arrested by FCS deprivation for 24 h in DMEM supplemented with sodium pyruvate (1 mm), l-glutamine (2 mm), nonessential amino acids (1:100), penicillin (100 units/ml)/streptomycin (100 μg/ml), amphotericin B (1.5 μg/ml), and bovine serum albumin (0.1%) (All from Sigma-Aldrich).

Cells were pretreated with JQ1/SGCBD01 (denoted as JQ1^+^) (3–1000 nm), its enantiomer (JQ1^−^) (3–1000 nm), or I-BET762 (3–1000 nm) (all from Tocris, Bristol, UK) or a c-Myc inhibitor (25–150 μm) (10058-F4; Sigma-Aldrich) for 1 h before being stimulated with 2.5% FCS ± TGF-β (1 ng/ml) (Sigma-Aldrich) at 8 days. We have previously demonstrated that this is the optimum time point to observe differences in phenotype between these ASM cells (4). Cell proliferation was measured by the cell proliferation ELISA BrdU kit (Roche Diagnostics) an assay comparable with measuring cell numbers as confirmed by flow cytometry (4, 28). Supernatants were removed, and IL-6 and CXCL8 levels were determined by DuoSet ELISA (R&D Systems, Abingdon, UK). IL-6 and CXCL8 levels were normalized to cell numbers, by dividing by the fold-change as measured by the BrdU assay. Cell viability was measured by 3-(4,5-dimethylthiazol-2-yl)-2,5-diphenyltetrazolium bromide (MTT) assay (29).

##### Transfection with siRNAs to Target Brd4 and c-MYC

ASM cells were transfected as previously described (4). Brd4 and c-MYC siRNA were obtained from (Thermo Scientific, Epsom, UK). Control siRNA was obtained from Ambion/Applied Biosystems (Paisley, UK). Transfected cells were plated into 96-well plates and left to adhere overnight before being serum starved for 6 h before stimulation with 2.5% FCS and 1 ng/ml TGF-β for 8 days.

##### mRNA Expression

*IL6*, *IL8*, *p21^WAF1^*, and *p27^kip1^* mRNA expression levels were measured as previously described (4, 30–32). In brief, total RNA was isolated using the RNeasy mini kit (Qiagen), and mRNA expression levels were determined using semiquantitative two-step RT-PCR using Assay on Demand primer/probe sets obtained from Applied Biosystems at 24 h. We have previously demonstrated that this is the optimum time point to observe differences in mRNA expression between these ASM cells (4).

##### Chromatin Immunoprecipitation Analysis

ChIP analysis was carried using the Magna ChIP^TM^ A/G chromatin immunoprecipitation kit following the manufacturer's instructions (Merck Millipore, Watford, UK). In brief, ASM cells were grown in culture and treated as described above. Following stimulation the cells were formalin-fixed. The cells were sonicated to shear DNA (5 × 15 s at 40% amplitude) to ∼200–1000 bp in length. Brd4 and c-Myc were immunoprecipitated using 1 μg of antibody (Santa Cruz Biotechnology, Santa Cruz, CA). After reverse cross-linking, the DNA was purified and quantitative PCR carried out using SybrGreen (Qiagen) and the following primers: pCXCL8 forward, 5′-GGGCCATCAGTTGCAAATC-3′; pCXCL8 reverse, 5′-TCCTTCCGGTGGTTTCTTC-3′; pIL6 forward, 5′-CAAGCCTGGGATTATGAAGAAGG-3′; and pIL6 reverse, 5′-AGCACTGGCAGCACAAGGCAAAC-3′. The amount of DNA recovered by ChIP was normalized to an input control. IgG isotype antibodies (provided by Millipore) were used as a negative control for the ChIP assay.

##### Data Analysis

The data were analyzed using GraphPad Prism version 5.03 (GraphPad Software, San Diego, CA). The data were not normally distributed (as assessed by the Kolmogorov-Smirnov test), and groups were compared using the Dunn's nonparametric test. Significance was defined as a *p* < 0.05.

## RESULTS

### 

#### 

##### Effect of JQ1/SGCBD01 on FCS+TGF-β-induced ASM Proliferation

**FIGURE 1.**
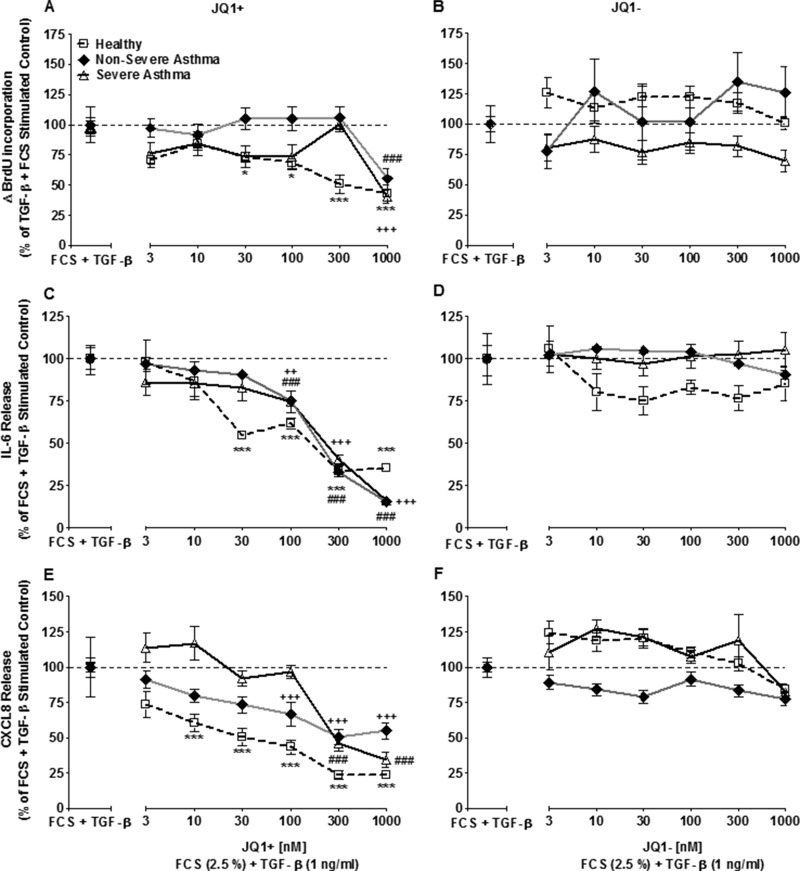
**JQ1/SGCBD01, but not its negative enantiomer (JQ1^−^), suppresses FCS and TGF-β-induced cell proliferation and cytokine release.** *A–F*, concentration-dependent effect of JQ1^+^/JQ1^−^ on FCS (2.5%) and TGF-β (1 ng/ml)-induced cellular proliferation (*A* and *B*), IL-6 release (*C* and *D*), and CXCL8 release (*E* and *F*) after 8 days. *Points* represent means ± S.E. from ASM cells from nine healthy (□*), nonsevere asthma (♦^#^), and severe asthma (▵^+^) subjects. *, *p* < 0.05; ***/###/+++, *p* < 0.001 compared with FCS+TGF-β-stimulated cells.

As previously reported, there was no difference in baseline proliferation rate between the cells from the three subject groups (4). Pretreatment of cells from healthy subjects with JQ1^+^ (30–1000 nm) inhibited FCS+TGF-β-induced proliferation in a concentration-dependent manner reaching a maximal effect of 43.3 ± 26.5% (*p* < 0.001) at 1000 nm (Fig. 1*A*). However, there was a reduced sensitivity to JQ1^+^ in ASM cells from both the nonsevere and severe asthmatic patients compared with that seen in ASM cells from healthy subjects in that only the highest concentration of JQ1^+^ (1000 nm) reduced cell proliferation (*p* < 0.001) (Fig. 1*A*). In contrast, the negative enantiomer, JQ1^−^, had no significant effect on proliferation of ASM cells from any patient group (Fig. 1*B*). In addition, neither JQ1^+^ nor JQ1^−^ showed any effect on ASM cell viability at any concentration tested (data not shown).

##### Effect of JQ1/SGCBD01 on FCS+TGF-β-induced IL-6 and CXCL8 Release

Pretreatment of cells from healthy subjects with JQ1^+^ (30–1000 nm) inhibited FCS+TGF-β-induced IL-6 release in a concentration-dependent manner by up to 35.2 ± 7.8% at 1000 nm in ASM cells from healthy individuals (*p* < 0.001; Fig. 1*C*). Although the asthmatic group appears to show reduced sensitivity and higher maximum effect of inhibitors, this is because the initial increases in both proliferation and cytokine release from the different patient subsets were normalized to 100%.

Similar to the results reported for proliferation, cells from patients with severe and nonsevere asthma had a reduced sensitivity to JQ1^+^ with a significant repression of IL-6 release observed at 100–1000 nm of JQ1^+^ (*p* < 0.001; Fig. 1*C*). The negative enantiomer, JQ1^−^, had no effect upon IL-6 release (Fig. 1*D*).

Although there was a concentration-dependent inhibition of FCS+TGF-β-induced CXCL8 release from cells from all groups, the effect of JQ1^+^ upon CXCL8 release was different from that seen with IL-6 release. Although 10 nm JQ1^+^ was sufficient to inhibit CXCL8 release from ASM cells isolated from healthy individuals (*p* < 0.001), 100–1000 nm JQ1^+^ was required to inhibit CXCL8 release from the nonsevere asthmatic ASM cells (*p* < 0.001), and only the highest concentrations of JQ1^+^ (300–1000 nm) inhibited CXCL8 release from the severe asthmatic ASM cells (*p* < 0.001) (Fig. 1*E*). No effect of the negative enantiomer, JQ1^−^, on CXCL8 release was observed (Fig. 1*F*).

##### Effect of I-BET762 on FCS+TGF-β-induced ASM Proliferation and Inflammation

**FIGURE 2.**
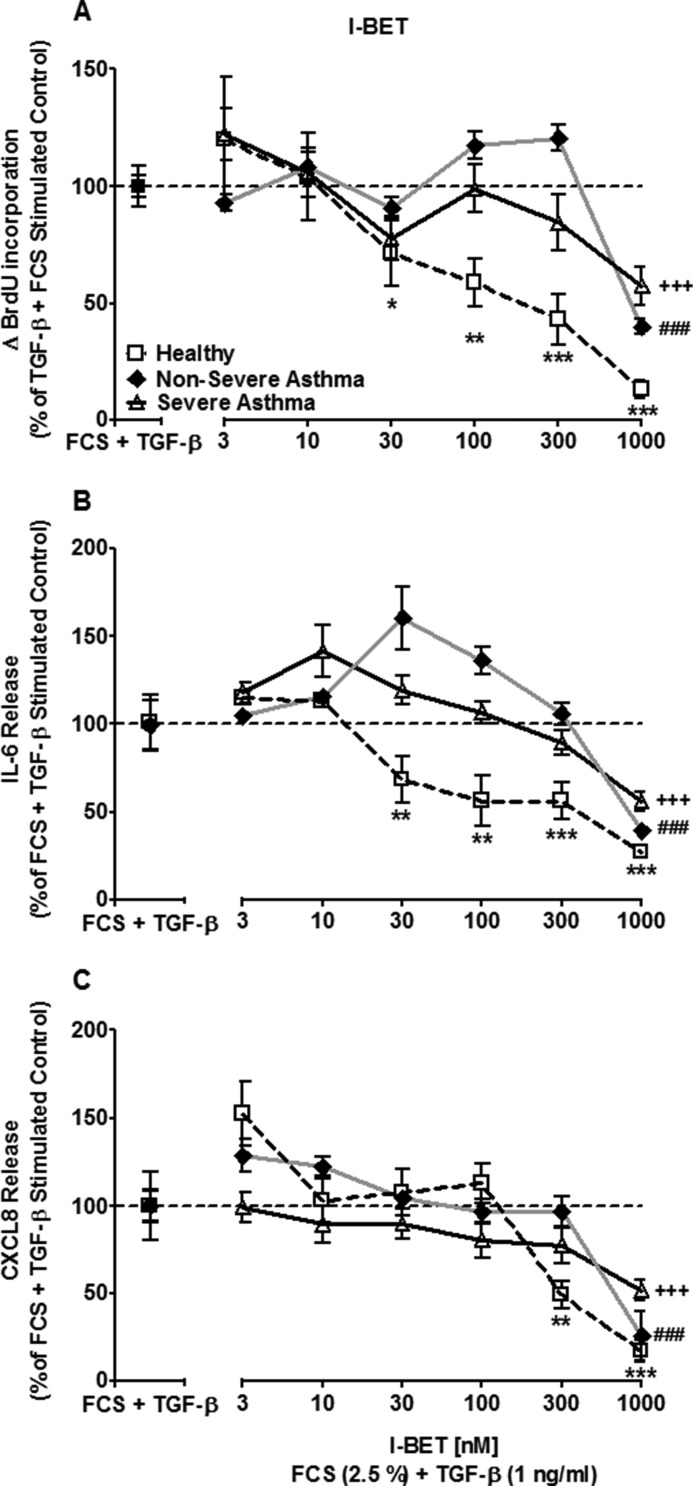
**I-BET762 suppresses FCS and TGF-β-induced cell proliferation and cytokine release.** *A–C*, the concentration-dependent effect of I-BET762 on FCS (2.5%) and TGF-β (1 ng/ml)-induced cellular proliferation (*A*), IL-6 release (*B*), and CXCL8 release (*C*) after 8 days. *Points* represent means ± S.E. from ASM cells from nine healthy (□*), nonsevere asthma (♦^#^), and severe asthma (▵^+^) subjects. *, *p* < 0.05; **, *p* < 0.01; ***/###/+++, *p* < 0.001 compared with FCS+TGF-β-stimulated cells.

We observed effects similar to those with JQ1+ with the structurally distinct BET mimic, I-BET762. I-BET762 (30–1000 nm) inhibited FCS+TGF-β-induced proliferation by ∼100% in ASM cells from healthy individuals (*p* < 0.001; Fig. 2*A*). However, only the highest concentration of I-BET762 (1000 nm) reduced FCS+TGF-β-induced proliferation in the ASM cells from both nonsevere and severe asthmatic patients (*p* < 0.001; Fig. 2*A*). No effect upon cell viability was seen at any concentration (data not shown).

I-BET762 (30–1000 nm) inhibited FCS+TGF-β-induced IL-6 release in a concentration-dependent manner by up to 27.2 ± 3.3% in ASM cells isolated from healthy individuals (*p* < 0.001; Fig. 2*B*). In contrast, inhibition of FCS+TGF-β-induced IL-6 release was seen with 1000 nm I-BET762 in ASM cells from both nonsevere and severe asthmatic patients (*p* < 0.001; Fig. 2*B*). FCS+TGF-β-induced CXCL8 release also had a different pattern of suppression by I-BET762 compared with that seen with IL-6 release. Suppression of FCS+TGF-β-induced CXCL8 release required an I-BET762 concentration of 300 nm or greater in healthy ASM cells (*p* < 0.01), whereas significant suppression of CXCL8 release was only seen at 1000 nm I-BET762 in cells from nonsevere and severe asthmatics (*p* < 0.001; Fig. 2*C*).

##### Effect of c-Myc Inhibition on FCS+TGF-β-induced Proliferation and Inflammation

**FIGURE 3.**
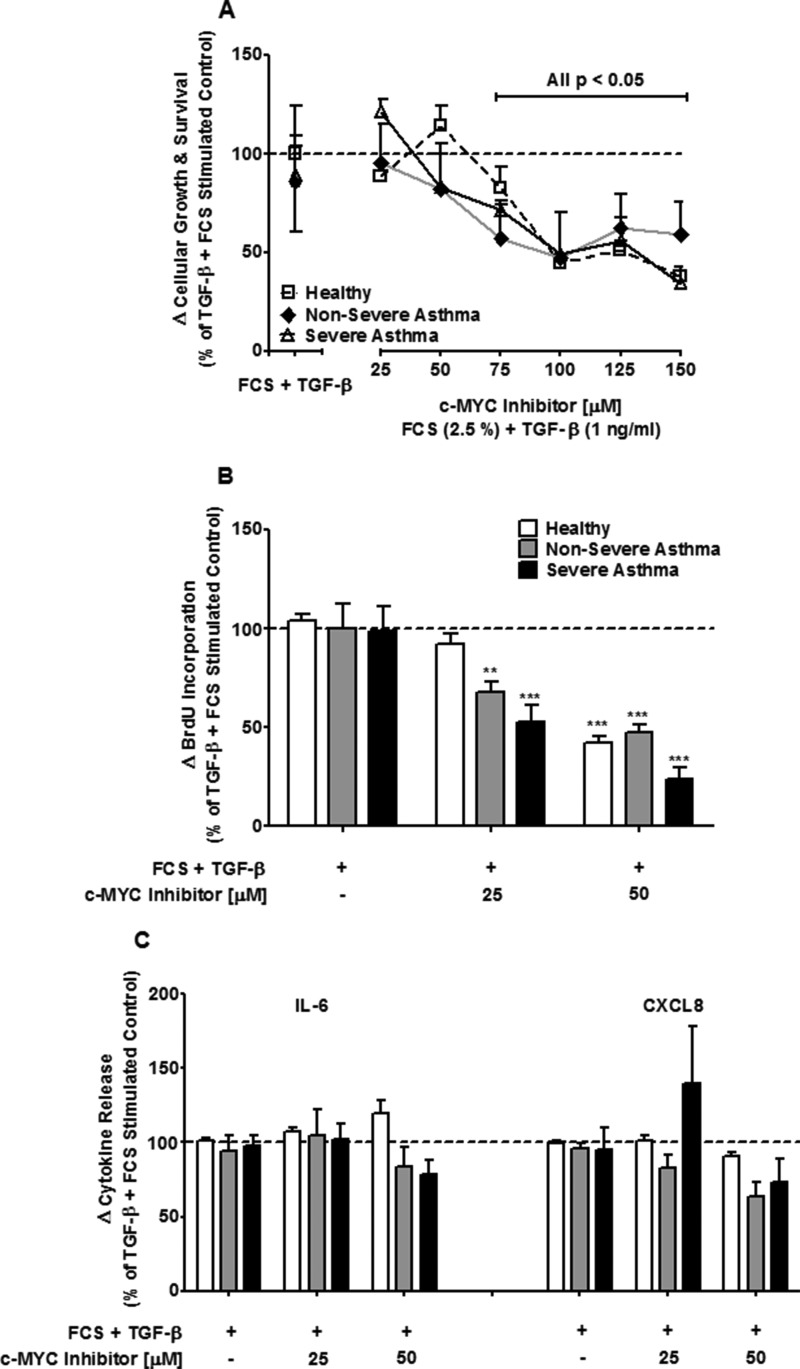
**Inhibition of c-Myc attenuates FCS and TGF-β-induced cell proliferation but not IL-6 and CXCL8 release.** *A–C*, concentration-dependent effect of the c-Myc inhibitor (10058-F4, 0–150 μm) on FCS (2.5%) and TGF-β (1 ng/ml)-induced cell viability (*A*), cellular proliferation (*B*), and IL-6 and CXCL8 release (*C*) from ASM cells from healthy (□), nonsevere asthma (♦), and severe asthma (▵) subjects at 8 days. *Bars*/*points* represent means ± S.E. from nine ASM donors in each group. *, *p* < 0.05; **, *p* < 0.01; ***, *p* < 0.001 compared with untreated cells.

To determine whether the effects of JQ1^+^ result from targeting the pro-oncogenic transcription factor, c-Myc, we analyzed the effect of the c-Myc inhibitor, 10058-F4, on proliferation and inflammation. Using a concentration range used in previous studies (33, 34), we found that concentrations higher than 75 μm decreased ASM growth and survival up to ∼50% in the ASM from all three groups (*p* < 0.05) (Fig. 3*A*). Therefore, we used the concentrations of 25 and 50 μm to examine effects on proliferation. 25 μm 10058-F4 inhibited FCS+TGF-β-induced proliferation in ASM cells from nonsevere (70.0 ± 10.7%) and severe asthmatics (52.9 ± 20.7%) (*p* < 0.01 and *p* < 0.001, respectively) (Fig. 3*B*). At the higher concentration of 50 μm, 10058-F4 inhibited proliferation in all three groups (all *p* < 0.001; Fig. 3*B*). 10058-F4 had no significant effect upon either IL-6 or CXCL8 release (Fig. 3*C*).

##### Effect of Brd4 and c-MYC Knockdown with siRNA on FCS+TGF-β-induced ASM Proliferation and Inflammation

**FIGURE 4.**
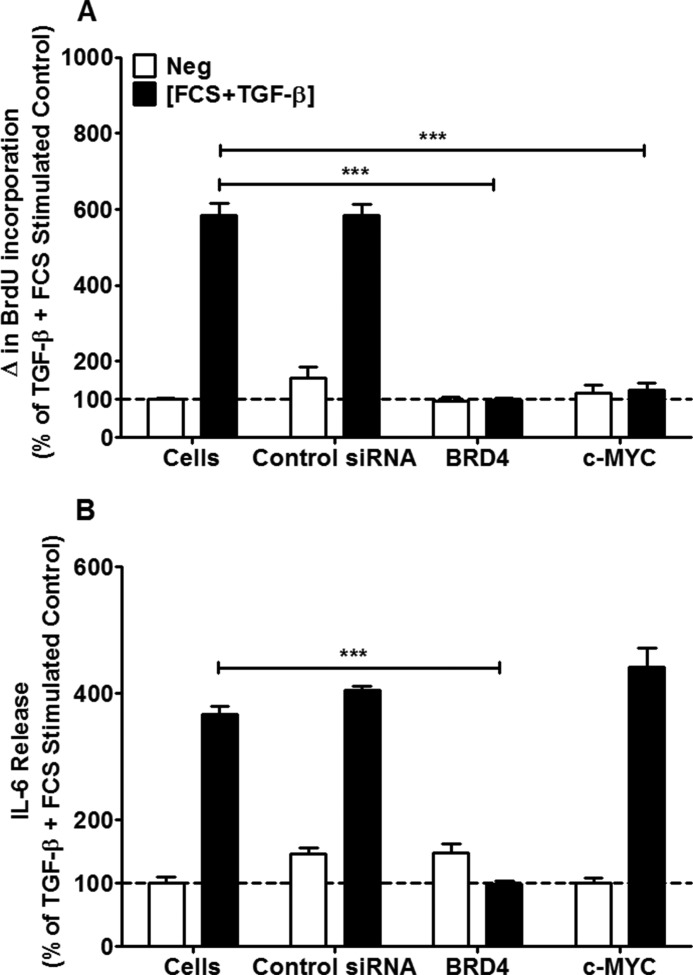
**Effect of targeting of Brd4 and c-MYC with siRNAs on FCS and TGF-β-induced cell proliferation and IL-6 release.** ASM cells from healthy subjects were transfected with siRNAs designed to target Brd4 and c-MYC, or a negative (*Neg*) control (100 nm), before being stimulated with FCS (2.5%) and TGF-β (1 ng/ml). Cellular proliferation (*A*) and IL-6 release (*B*) were measured at 8 days. *Bars* represent means ± S.E. from nine ASM donors. ***, *p* < 0.001 compared with stimulated cells.

To confirm the above results with pharmacological tools targeting c-Myc and Brd4, we then transfected siRNAs into the healthy ASM cells designed to target Brd4 and c-MYC. Inhibition of Brd4 with 300 nm of siRNA inhibited both the FCS+TGF-β-induced ASM proliferation and IL-6 release (*p* < 0.001) (Fig. 4, *A* and *B*). However, siRNA targeted inhibition of c-MYC (300 nm) only resulted in a reduction in ASM proliferation (Fig. 4*A*) (*p* < 0.001) and not IL-6 release (Fig. 4*B*).

##### Effect of JQ1/SGCBD01 on c-MYC, p21^WAF1^, and p27^kip1^ mRNA in ASM

**FIGURE 5.**
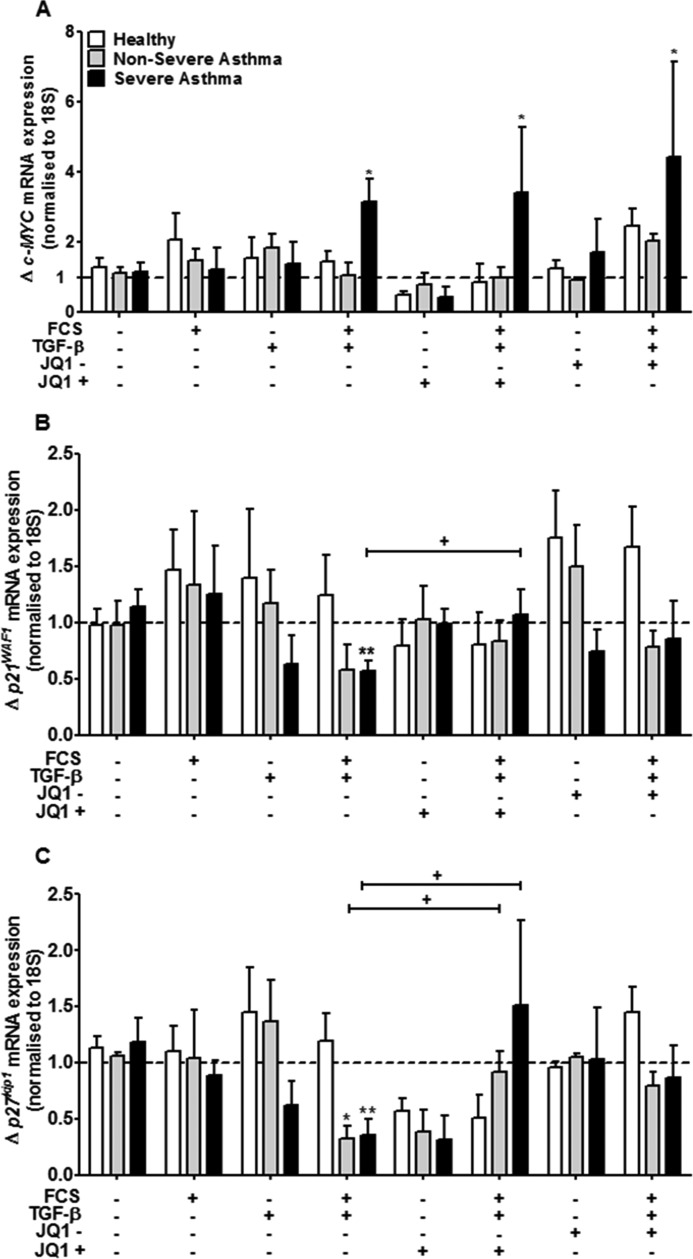
**Effect of JQ1/SGCBD01 on FCS and TGF-β-induced *c-MYC*, *p21*WAF1 and *p27*kip1 mRNA expression.** *A–C*, effect of JQ1^+^ and JQ1^−^ pretreatment (both at 1 μm) on FCS (2.5%) and TGF-β (1 ng/ml)-induced *c-MYC*, (*A*), *p21^WAF1^* (*B*), and *p27^kip1^* (*C*) mRNA expression in ASM cells from healthy, nonsevere asthma and severe asthma subjects at 24 h. *Bars* represent means ± S.E. from nine ASM donors in each group. *, *p* < 0.05; **, *p* < 0.01; ***, *p* < 0.001 compared with unstimulated cells. +, *p* < 0.05; +++, *p* < 0.001 compared with FCS+TGF-β-stimulated cells.

We next examined the effect of JQ1^+^ upon *c-MYC* mRNA expression. We found that FCS+TGF-β increased the expression of *c-MYC* in ASM cells from severe asthmatics by 2.2 ± 1.6-fold, with no effect on ASM cells from normal and nonsevere asthmatics (*p* < 0.05) (Fig. 5*A*). Neither JQ1^+^ nor JQ1^−^ had any effect on this increased expression (Fig. 5*A*).

We also examined the effect of JQ1^+^ upon *p21^WAF1^* and *p27^kip1^* mRNA levels. We have previously demonstrated that these cyclin inhibitors are crucial for ASM proliferation (7). As previously seen, we showed a decrease in *p21^WAF1^* mRNA expression following FCS+TGF-β stimulation in the ASM cells from the severe asthmatics by 0.53 ± 0.32-fold (*p* < 0.01) (Fig. 5*B*). There was a decrease in *p27^kip1^* expression in both the nonsevere (0.46 ± 0.30, *p* < 0.05) and severe (0.29 ± 0.31, *p* < 0.01) ASM cells (Fig. 5*C*). JQ1^+^ recovered the expression of both *p21^WAF1^* in the severe asthmatics (*p* < 0.05) (Fig. 5*B*) and *p27^kip1^* in both the nonsevere and severe asthma subjects back to basal levels (both *p* < 0.05) (Fig. 5*C*).

##### Effect of JQ1/SGCBD01 on IL6 mRNA in ASM and upon Binding of Brd4 to the IL6 Gene Promoter

**FIGURE 6.**
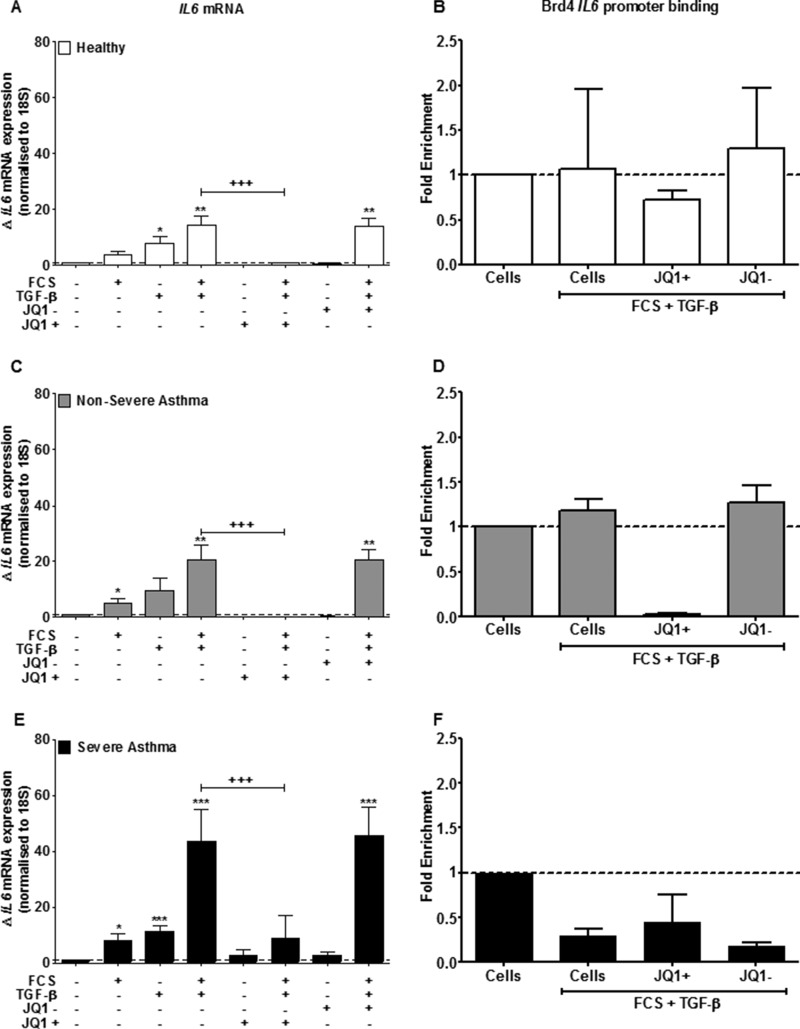
**JQ1/SGCBD01 attenuates FCS and TGF-β-induced *IL6* mRNA expression and decreases association with *IL6* promoters.** *A*, *C*, and *E*, effect of JQ1^+^ and JQ1^−^ pretreatment (both at 1 μm) on FCS (2.5%) and TGF-β (1 ng/ml)-induced *IL6* mRNA expression in ASM cells from healthy (*A*), nonsevere asthma (*C*), and severe asthma (*E*) subjects at 24 h. *Bars* represent means ± S.E. from nine ASM donors in each group. *B*, *D*, and *F*, effect of JQ1^+^ and JQ1^−^ on FCS (2.5%) and TGF-β (1 ng/ml)-induced Brd4 *IL6* promoter binding measured by ChIP in ASM cells from healthy (*B*), nonsevere asthma (*D*), and severe asthma (*F*) subjects at 24 h. *Bars* represent means ± S.E. from three ASM donors in each group. *, *p* < 0.05; **, *p* < 0.01; ***, *p* < 0.001 compared with unstimulated cells. +++, *p* < 0.001 compared with FCS+TGF-β-stimulated cells.

To determine whether BET regulation of IL-6 release occurred at the transcriptional level, we analyzed the expression of *IL6* mRNA at 24 h poststimulation. Both FCS and TGF-β alone induced a small increase in *IL6* mRNA in all cell types, although the response was greater in cells from severe asthmatics (*p* < 0.001) (Fig. 6, *A*, *C*, and *E*).

FCS+TGF-β in combination induced a significant induction of *IL6* mRNA in cells isolated from healthy individuals (14.3 ± 8.9, *p* < 0.01) (Fig. 6*A*), nonsevere asthmatics (20.6 ± 12.6, *p* < 0.01) (Fig. 6*C*), and severe asthmatics (43.7 ± 27.4, *p* < 0.01) (Fig. 6*E*). There was a greater response in cells from severe asthmatics compared with normal (*p* < 0.01). When these cells were pretreated with JQ1^+^ (1000 nm) for 1 h before FCS+TGF-β stimulation, there was almost complete suppression of *IL6* mRNA expression in all patient groups (*p* < 0.001) (Fig. 6, *A*, *C*, and *E*). The negative enantiomer, JQ1^−^, had no effect upon the transcription of *IL6*.

We used ChIP analysis to investigate the binding of Brd4 to the *IL6* promoter region. ASM cells were serum-starved for 24 h before being stimulated with FCS (2.5%) and/or TGF-β (1 ng/ml) for 24 h. In some cases, cells were pretreated for 1 h with either 1000 nm JQ1^+^ or JQ1^−^. There was more Brd4 associated with the *IL6* gene promoter in the ASM cells from the severe asthmatics compared with controls at baseline (data not shown). Although the ChIP experiments did not show significance, there was a trend for increased Brd4 association with the *IL6* promoter with FCS+TGF-β treatment in the healthy subjects and nonsevere asthmatics, which was reduced by JQ1^+^ but not by JQ1^−^ (Fig. 6, *B* and *D*). Furthermore, stimulation of severe asthmatic ASM cells with FCS+TGF-β reduces fold enrichment of *IL6*. The addition of JQ1^+^ to these same cells increases this reduction in enrichment, and JQ1^−^ has no effect (when comparing to the *Cells* + *[FCS*+*TGF*-β*]* bar). Importantly, we were unable to show clear evidence for c-Myc association with the *IL6* promoters in ASM cells from healthy subjects or asthmatic subjects compared with control input samples and no evidence for enhanced c-Myc binding following stimulation of the cells with FCS (2.5%) and/or TGF-β (1 ng/ml) in the presence or absence of JQ1^+^ or JQ1^−^ (data not shown).

##### Effect of JQ1/SGCBD01 on CXCL8 mRNA in ASM and upon Binding of Brd4 to the IL8 Gene Promoter

**FIGURE 7.**
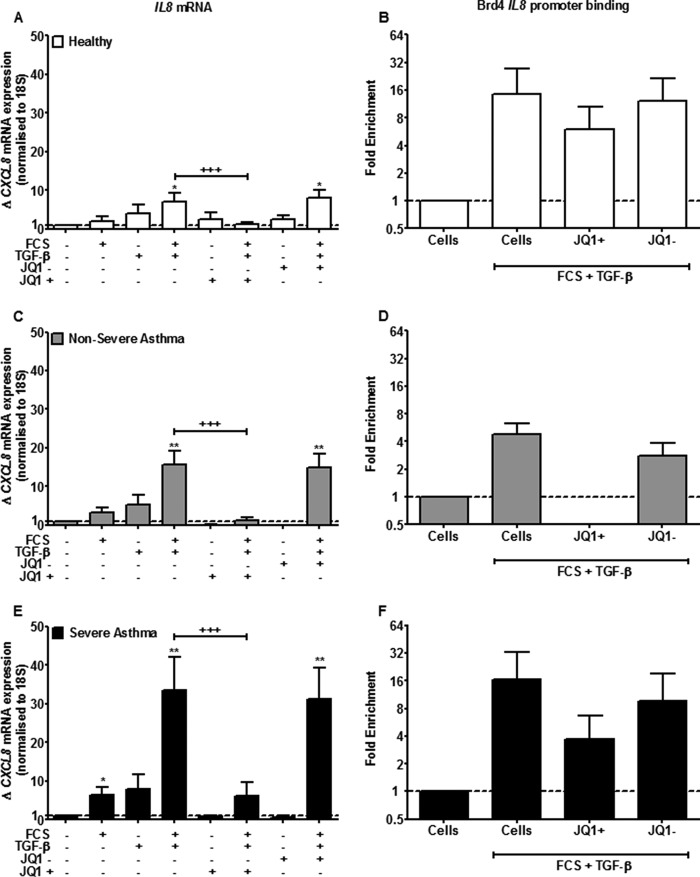
**JQ1/SGCBD01 attenuates FCS and TGF-β-induced *IL8* mRNA expression and decreases association with *IL8* promoters.** *A*, *C*, and *E*, effect of JQ1^+^ and JQ1^−^ pretreatment (both at 1 μm) on FCS (2.5%) and TGF-β (1 ng/ml)-induced *IL8* mRNA expression in ASM cells from healthy (*A*), nonsevere asthma (*C*), and severe asthma (*E*) subjects at 24 h. *Bars* represent means ± S.E. from nine ASM donors in each group. *B*, *D*, and *F*, effect of JQ1+ and JQ1- on FCS (2.5%) and TGF-β (1 ng/ml)-induced Brd4 IL8 promoter binding measured by ChIP in ASM cells from healthy (*B*), nonsevere asthma (*D*), and severe asthma (*F*) subjects at 24 h. *Bars* represent means ± S.E. from three ASM donors in each group. *, *p* < 0.05; **, *p* < 0.01 compared with unstimulated cells. +++, *p* < 0.001 compared with FCS+TGF-β-stimulated cells.

Similarly to IL-6, both FCS and TGF-β alone induced a small increase in *IL8* mRNA in all cell types, although the response was always greater in cells from severe asthmatics (*p* < 0.001) (Fig. 7, *A*, *C*, and *E*).

FCS+TGF-β in combination induced a significant induction of *IL8* mRNA in cells isolated from healthy individuals (7.0 ± 5.4, *p* < 0.05), nonsevere asthmatics (15.6 ± 8.9, *p* < 0.01), and severe asthmatics (33.4 ± 21.4, *p* < 0.01) (Fig. 7, *A*, *C*, and *E*). There was a greater response in cells from severe asthmatics compared with normal (*p* < 0.01). When these cells were pretreated with JQ1^+^ (1000 nm) for 1 h before FCS+TGF-β stimulation, there was almost complete suppression of *IL8* mRNA expression in all patient groups (*p* < 0.001) (Fig. 7, *A*, *C*, and *E*). The negative enantiomer, JQ1^−^, had no effect upon the transcription of *IL8.*

Once again, we used ChIP analysis to investigate the binding of Brd4 to the *IL8* promoter region. Similarly to *IL6*, there was more Brd4 associated with the *IL8* gene promoter in the ASM cells from the severe asthmatics compared with controls at baseline (data not shown). There was a trend for increased Brd4 association with the *IL8* promoter with FCS+TGF-β treatment in all subjects, which was reduced by JQ1^+^ but not JQ1^−^ (Fig. 7, *B*, *D*, and *F*). There was no clear evidence for c-Myc association with the *IL8* promoter in ASM cells from any subjects compared with control input samples and no evidence for enhanced c-Myc binding following stimulation of the cells with FCS (2.5%) and/or TGF-β (1 ng/ml) in the presence or absence of JQ1^+^ or JQ1^−^ (data not shown).

## DISCUSSION

We have shown that the BET mimics JQ1/SGCBD01 and I-BET762 inhibited FCS+TGF-β-induced cell proliferation and inflammation in ASM cells from healthy and asthmatic individuals. The concentrations required to inhibit proliferation were higher in cells from nonsevere and severe asthmatics, indicating that there is an innate resistance of asthmatic cells to the effect of these BET mimics. Inhibition of c-Myc affected ASM proliferation but not IL-6 or CXCL8 production. JQ1/SGCBD01 reduced the association of Brd4 to the *IL6* and *IL8* promoters, thereby modulating their transcription.

We showed that BET proteins in primary ASM cells are important in controlling ASM cell proliferation both in healthy and asthmatic cells. Previous studies have mainly focused on cancerous cell lines, where JQ1/SGCBD01 inhibited cellular proliferation of NUT midline carcinoma (35), lung cancer cells (36), acute myeloid leukemia cells (37), and a human leukemic cell line (UT7) (38). This mechanism is documented to occur via a decrease in either c-Myc expression or c-Myc association with a number of proliferative gene promoters. c-Myc is a regulatory transcription factor that regulates cell growth, and deregulation of c-Myc in cancer contributes to proliferation, tumorigenesis, and resistance to apoptosis. Furthermore, amplification of c-Myc is one of the most common genetic alterations in cancer genomes (39). Although we show that inhibition of c-Myc inhibits proliferation, we saw no effect of JQ1/SGCBD01 upon the induced expression of c-Myc expression by FCS+TGFβ, demonstrating that the effect of JQ1^+^ upon c-Myc expression seen in cancerous cells and nonprimary cells is different from that seen in noncancerous asthmatic ASM cells.

Our results provide evidence that the effect of JQ1/SGCBD01 and I-BET762 is independent of the induction of c-Myc, as has been demonstrated in glioblastomas where JQ1/SGCBD01 had a minimal effect upon c-Myc expression. JQ1/SGCBD01 had a significant effect upon *p21^WAF1^* expression, returning it to basal levels (35). We have previously shown that primary ASM proliferation is under the control, at least in part, of *p21^WAF1^* and *p27^kip1^* (4, 6). Here we show that JQ1/SGCBD01 may mediate its response in asthmatic ASM by returning the reduced expression of these cyclin inhibitors to basal levels. The release of IL-6 and CXCL8 from ASM cells was not affected by pharmacological inhibition of c-Myc which corresponded with the failure to show marked physical association of c-Myc to the *IL6* and *IL8* promoters.

We also demonstrate that BET bromodomain mimics JQ1/SGCBD01 and I-BET762 inhibited FCS+TGF-β-induced release and gene expression of IL-6 and CXCL8 in ASM cells from all three groups of subjects. However, these inhibitors were less effective on ASM cells from patients with asthma. Our chromatin immunoprecipitation data show that there was a greater amount of enrichment of Brd4 at the *IL6* and *IL8* promoters at baseline in ASM cells from severe asthma compared with nonasthmatics subjects. FCS+TGF-β increased Brd4 binding to the *IL6* and *IL8* promoters in the controls but only the *IL8* promoter in the ASM cells isolated from severe asthmatics. JQ1/SGCBD01 did not significantly inhibit this enrichment of either the *IL6* or the *IL8* promoters.

Increased Brd4 binding at baseline may allow greater *IL6* and *IL8* gene expression, explaining the increased inflammation in severe asthmatics compared with controls. Thus, inhibition of Brd4 binding to the gene promoters may explain the mechanism of action of JQ1/SGCBD01 and by extension of I-BET762 in inhibiting cytokine gene expression. The differential effect of JQ1/SGCBD01 and I-BET762 on IL-6 and CXCL8 expression and on basal Brd4 promoter association may reflect differences in the underlying mechanisms controlling these genes, with *IL6* expression being regulated by Brd4 proteins at a paused promoter, whereas *IL8* transcription requires Brd4 recruitment before transcription can occur. Promoter pausing is an increasingly identified as a key regulatory step in the transcription of rapidly transcribed genes, including those involved in stress and inflammation (40, 41). Brd4 may be bound to the *IL6* promoter region prior to the TGF/FCS signal being received, allowing more rapid response to stimuli. Brd4 has previously been associated with recruitment of the pause release factor p-TEFB (42), and we therefore believe that the role of transcriptional pausing in the regulation of inflammation will make an interesting area of future research.

Similar findings have been reported with both JQ1/SGCBD01 and I-BET762. In murine bone marrow-derived macrophages exposed to bacterial endotoxin, I-BET762 inhibited the expression of pro-inflammatory cytokines such as IL-1β, IL-6, IL-12α, CXCL9, and CCL2, and I-BET762 displaced the BET proteins Brd2, Brd3, and Brd4 from the *IL6* promoter (23). I-BET762 was also shown to rescue mice from endotoxin-induced death. Similar results using JQ1/SGCBD01 were obtained for the endotoxin-induced production of IL-6 and TNF-α in mouse macrophages. In these experiments, Brd2 and c-Myc were shown to be physically associated with the promoter regions of these inflammatory genes (26). Both I-BET762 and JQ1/SGCBD01 have been reported to inhibit the release of IL-6 from human lung fibroblasts and their proliferation induced by TGF-β, by reducing Brd2 and Brd4 association with growth factor-responsive genes and enhanced transcription of these genes (43). The differential effect of JQ1/SGCBD01 and I-BET762 on IL-6 and CXCL8 expression and on basal Brd4 promoter association may reflect differences in the underlying mechanisms controlling these genes, with *IL6* expression being regulated by Brd proteins at a paused promoter, whereas *IL8* transcription requires Brd recruitment before transcription can occur.

A higher concentration of both JQ1/SGCBD01 and I-BET762 was needed to inhibit proliferation and IL-6 and CXCL8 release from cells from patients with asthma compared with nonasthmatic subjects. Asthmatic ASM cells demonstrate a greater degree of proliferation and release of cytokines such as IL-6 when exposed to FCS+TGF-β compared with ASM cells from nonasthmatic subjects (4, 15). The increased Brd4 association observed in the asthmatic samples (and possible transcriptional pausing of the inflammatory gene promoters) may explain the higher levels of transcription of *IL6* and *IL8* after stimulation of the cells (Figs. 6 and 7). This may also explain the need for a greater amount of the BET mimics to produce the same effect as in nonasthmatic ASM cells. Part of the explanation could lie with the greater association of Brd4 to the *IL6* promoter observed in severe asthmatics at baseline compared with controls at baseline. Although not significant, the binding of Brd4 to the promoter regions of *IL6* and *IL8* increased when the cells were stimulated by FCS+TGF-β, an effect that was prevented by pretreatment of the cells with JQ1/SGCBD01.

The relative insensitivity of ASM cells from asthma patients to the BET mimics reminds us of the similar insensitivity observed with corticosteroids (4, 15). The basis for corticosteroid insensitivity in asthma may be multifactorial (44). A lack of recruitment of histone deacetylase 2 to the RNA polymerase compatible with histone deacetylase activity being reduced in peripheral blood mononuclear cells and alveolar macrophages from patients with asthma has been proposed (18, 45). The gene promoters in cells from asthma *versus* nonasthma may have an enhanced acetylation status that may in part account for the reduced sensitivity to BET mimics in these cells.

BET bromodomain mimics can inhibit both aberrant ASM cell proliferation and pro-inflammatory cytokine production from cells from patients with asthma including severe asthma. Interestingly, a pharmaceutical company, Tensha Therapeutics, is currently in phase I clinical trials evaluating a derivative of JQ1/SGCBD01 (TEN-010) in patients with solid tumors (46), and although local delivery of these compounds has yet to be studied, systemic administration of JQ1/SGCBD01 significantly inhibits inflammatory cytokine expression in diseased gingival tissues (47) and demonstrates no affect upon systemic blood pressure (7). These compounds may therefore be effective in reducing features of airway remodeling in severe asthma, which often forms the basis of chronic airflow obstruction and greater inflammatory responses that are often insensitive to the effects of anti-inflammatory agents such as corticosteroids.

## Footnotes

4 The abbreviation used is:

ASM: airway smooth muscle.
